# Influence of Coastal Upwelling on SST Trends along the South Coast of Java

**DOI:** 10.1371/journal.pone.0162122

**Published:** 2016-09-08

**Authors:** R. Varela, F. Santos, M. Gómez-Gesteira, I. Álvarez, X. Costoya, J. M. Días

**Affiliations:** 1 Ephyslab, Environmental Physics Laboratory, Facultad de Ciencias, Universidad de Vigo, 32004 Ourense, Spain; 2 CESAM, Departamento de Física, Universidade de Aveiro, 3810–193 Aveiro, Portugal; University of California San Diego, UNITED STATES

## Abstract

The south coast of Java has warmed at a much lower rate than adjacent ocean locations over the last three decades (1982–2015). This behavior can be observed during the upwelling season (July-October) and it is especially patent in August and September when upwelling attains the highest values. Although different warming rates (ocean-coast) had been previously observed in other areas around the world, this behavior was always linked to situations where upwelling increased or remained unchanged. South Java warming is observed at ocean locations and cooling near shore but under a scenario of decreasing upwelling (~30% in some cases). The origin of coastal cooling is due to changes in the vertical structure of the water column. A vein of subsurface water, which has cooled at a rate higher than 0.3°C per decade, is observed to enter from the northwestern part of the study area following the South Java Current. This water only manifests at surface near coast, where it is pumped up by coastal upwelling.

## Introduction

Climate change is affecting a wide range of systems. In particular are important the impacts over the oceans which have been subject to global rising temperatures in the last decades [1–3]. Recently, Lima and Wethey [3] analyzed changes in coastal Sea Surface Temperature (SST) worldwide at a scale of 0.25° over the last three decades (1982–2010). They found that 71% of the world’s coastlines have been significantly warming and that rates of change have been highly heterogeneous in both space and season. Since upwelling systems are one of the most important spots of productivity it is also necessary to analyze in detail the evolution of SST patterns over these areas. Over the last years, several authors have described differences between warming rates at coastal and ocean locations in different coastal upwelling systems [4–8]. Thus, Lemos and Sanso [4] found a difference of 0.1°C dec^-1^ between coast and ocean locations along the western Iberian Peninsula using data with a spatial resolution of 0.25°×0.25° from 1901 to 2000. Similar results were obtained by Santos et al. [5] from 1958 to 2008 at a scale of 0.5°. In the Canary upwelling system, Santos et al. [6] detected an ocean warming rate higher than the coastal one (around 0.5°C dec^-1^) from 1982 to 2010 using high resolution SST data (4 x 4 km). Santos et al. [7] also observed weaker warming trends at coastal locations along the Benguela upwelling system from 1970 to 2009 considering data on a 1°x1° grid (difference ocean-coast ~0.4°C dec^-1^). More recently, Santos et al. [8] described the existence of a difference around 0.15–0.2°C dec^-1^ between coast and ocean locations along La Guajira upwelling system from 1982–2014 at a scale of 0.25°. These differences between coast and ocean along the different upwelling systems can be linked to the strengthening of coastal upwelling also detected by some of the previous authors. Recently, Varela et al [9] analyzed trends in the coastal upwelling regime worldwide using wind data over the last three decades. They also found increasing trends in upwelling in the coastal areas of Benguela and Canary. These results suggest the role of upwelling as a moderator of general SST increase in coasts affected by this mechanism. This fact shows the interest to direct the spotlight towards other local areas of the world where upwelling is also an important forcing as, for example, the south coast of Java.

Java is located in the Eastern Indian Ocean where the southeast (SE) monsoon dominates during the austral winter [10–15]. This SE monsoon generates seasonal upwelling which produces cold water in the surface, creating a difference on SST and biological productivity between coastal and ocean-ward locations [13, 16–20]. During the rest of the year, westerly winds linked to the west monsoon arise generating periods of weak and variable winds without upwelling productivity.

Upwelling along the south coast of Java has been mainly characterized in terms of upwelling occurrence. This coast presents a constant E-W orientation, showing a different behavior between the eastern and western coast. The western zone usually presents more intense zonal winds while coastal upwelling is stronger in the eastern area [15, 21]. Different researchers have found that upwelling starts in the eastern area and move towards the equator [10, 11, 16, 22–24]. Lower temperature values were also observed along the eastern area associated with strong upwelling [19, 21].

Upwelling trends along this coast have only been recently analyzed by Varela et al. [9] in terms of wind stress at a scale of 0.3°. They described a significant negative trend for the entire coast during the considered upwelling season (May-October) from 1982 to 2010. On the other hand, Lima and Wethey [3] estimated changes in coastal SST along this coast over the same period of time (1982–2010) at a scale of 0.25°. These authors found a general coastal warming almost throughout the year except from June to September mainly at the western area where negligible and even small negative trends were detected. Considering the results of these two studies, a decrease in upwelling favorable winds and a small coastal cooling was detected along the south coast of Java over the last three decades. To investigate these observed patterns, coastal upwelling and SST trends along this coast will be analyzed in detail during the upwelling season.

The aim of this paper was to analyze SST trends along the south Java coast and its relation with upwelling during the last 34 years of strong climate change using high spatial resolution data. Differences in the warming rates between coastal and oceanic locations were also characterized to examine the role played by coastal upwelling. For this purpose, daily SST and wind data at a scale of 0.25° and 0.3°, respectively, were evaluated during the upwelling season defined from July to October. Information about the vertical structure of temperature was also provided considering data from the Simple Ocean Data Assimilation (SODA) project with a horizontal resolution of 0.5° and a vertical resolution of 40 levels.

## Material and Methods

**Fig 1:**
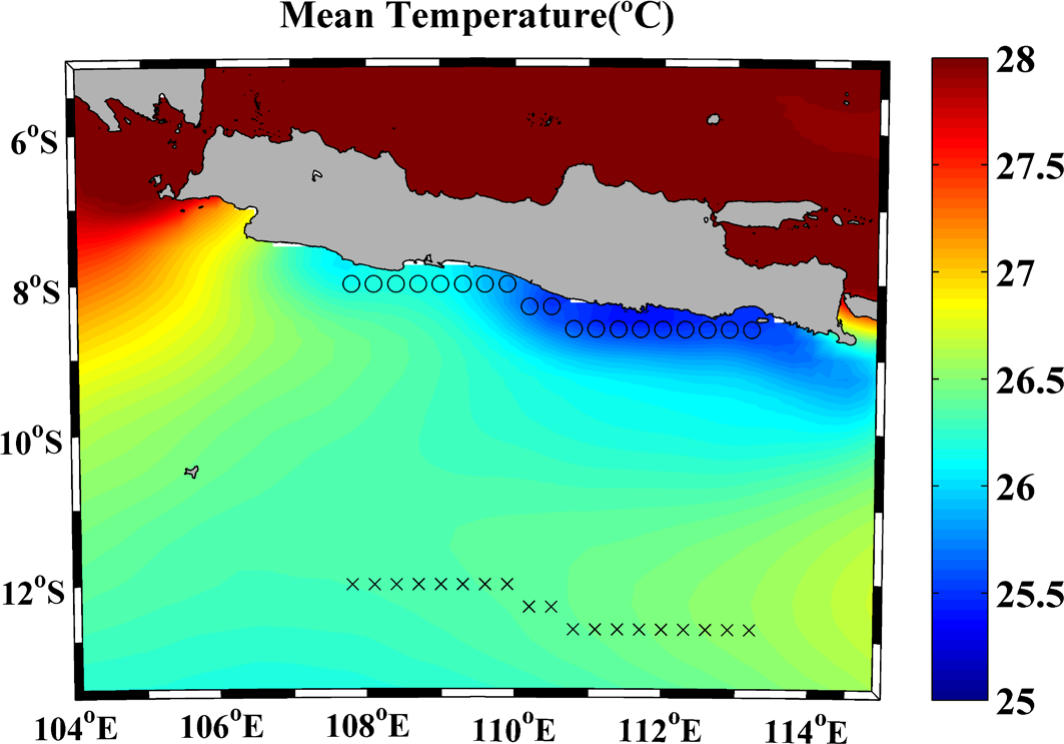
Mean temperature (°C) calculated from 1982 to 2015 for the upwelling season (July to October) using OISST ¼ database. Circles and crosses mark points where wind and SST data from CFSR and OISST ¼ were considered.

The area under study is the south coast of Java. Fig 1 shows the SST mean for the entire region over the period 1982–2015 from July to October (upwelling season). SST differences can be observed between the western and eastern coastal areas with the lowest temperature values at the easternmost zone. Differences between coast and ocean are also observed with SST increasing seaward. The presence of cooler surface water along the coast reveals the existence of coastal upwelling. Thus, to analyze in detail SST trends and its relation with upwelling, the area under study was focused on the central region of the southern Java coast from 107° to 113°E. Circles and crosses in Fig 1 represent the points under study in terms of Upwelling Index (UI) and SST data.

### Wind and Heat Fluxes Data

Wind and heat fluxes data were acquired from the NCEP CFSR database at http://rda.ucar.edu/pub/cfsr.html developed by the National Oceanic and Atmospheric Administration (NOAA). Data were obtained from the NOAA National Operational Model Archive and Distribution System, which is supported by the NOAA National Climatic Data Center. Additional information about the CFSR database can be found in Saha et al. [25]. The spatial resolution is 0.3°×0.3° from January 1982 to April 2011 and 0.2°×0.2° from then on. Data from the latter period were interpolated on a 0.3°×0.3° grid to use a common resolution over the whole period. Wind is calculated at a reference height of 10 m with 6-hourly time resolution.

Coastal upwelling analysis needs the use of pixels as close to shore as possible to represent coastal processes. To avoid problems with land contamination, only coastal pixels with less than 25% of land were used.

Daily wind data were averaged at monthly scale in order to calculate wind module using the equation: $|W|={\left(W_{x}^{2}+W_{y}^{2}\right)}^{1/2}$ where *W*_*x*_ is the zonal wind component and *W*_*y*_ is the meridional wind component.

Ekman Transport components were calculated as follows:

$$Q_{x}=\frac{\rho_{a}C_{d}}{\rho_{w}f}{\left(W_{x}^{2}+W_{y}^{2}\right)}^{\frac{1}{2}}W_{y}$$ (1)

$$Q_{y}=-\frac{\rho_{a}C_{d}}{\rho_{w}f}{\left(W_{x}^{2}+W_{y}^{2}\right)}^{\frac{1}{2}}W_{x}$$ (2)

where *ρ*_*w*_ = 1025*Kgm*^−3^ is the sea water density, *C*_*d*_ = 1.4 × 10^−3^ the drag coefficient, *ρ*_*a*_ = 1.22*Kgm*^−3^ the air density and f is the Coriolis parameter defined as *f* = 2*Ω*sin(*θ*) where *Ω* is the angular velocity and *θ* is the latitude.

UI is defined as the Ekman transport component in the direction perpendicular to the shoreline as follows [26]:

$$UI=-\sin\left(\theta -\frac{\pi }{2}\right)Q_{x}+\cos\left(\theta -\frac{\pi }{2}\right)Q_{y}$$ (3)

where θ is the angle of the unitary vector perpendicular to the coastline pointing oceanward. In this study, angles ranged from 250° to 270°. Positive (negative) upwelling indices correspond to upwelling-favorable (unfavorable) conditions.

Heat fluxes (shortwave, longwave, latent and sensible) were also obtained from the CFSR database at monthly scales. The net heat flux (*Q*_*T*_) through the ocean surface was calculated following Eq (4):

$$Q_{T}=Q_{SW}+Q_{LW}+Q_{S}+Q_{L}$$ (4)

where Q_SW_ is the shortwave flux, Q_LW_ is the longwave flux, Q_S_ is the sensible heat flux and Q_L_ is the latent heat flux. A negative (positive) heat flux implies that ocean is losing (gaining) heat.

### Temperature data

Daily SST values were obtained from the Optimum Interpolation Sea Surface Temperature (OISST) ¼ database. This database uses Advanced Very High Resolution radiometer (AVHRR) infrared satellite SST data and data from ships and buoys to build a regular global grid (more information can be found in Reynolds [27] and Reynolds and Chelton [28]). Daily files with a spatial resolution of 0.25°×0.25° were obtained from the NOAA website (http://www.ndc.noaa.gov/sst/). Daily SST values were averaged at monthly scale in order to calculate SST assuming linear regression.

Sea Temperature data beneath the sea surface were also obtained from the Simple Ocean Data Assimilation (SODA). This project has reanalyzed data from different sources (oceanographic cruises, satellite, model simulations). Reanalysis data are available at monthly scale with a horizontal resolution of 0.5°×0.5° and a vertical resolution of 40 levels (http://apdrc.soest.hawaii.edu/las/v6/dataset?catitem=3273) from 1958 to 2010. Detailed information about the methodology can be found in Carton et al. [29–30].

### Upwelling index, heat flux and Temperature trends

Trends were calculated at each pixel as the slope of the linear regression of UI, heat flux and temperature anomalies versus time. Monthly anomalies were calculated by subtracting from the UI, heat flux and temperature of a certain month the mean UI, heat flux and temperature of that month over the period 1982–2015. All trends were calculated using raw data without any filter or running mean. The Spearman rank correlation coefficient was used to analyse the significance of trends due to its robustness to deviations from linearity and its resistance to the influence of outliers. The significance level of each pixel is shown in the figures for those points that exceed 90% (circle) or 95% (square) of significance.

## Results and Discussion

**Fig 2:**
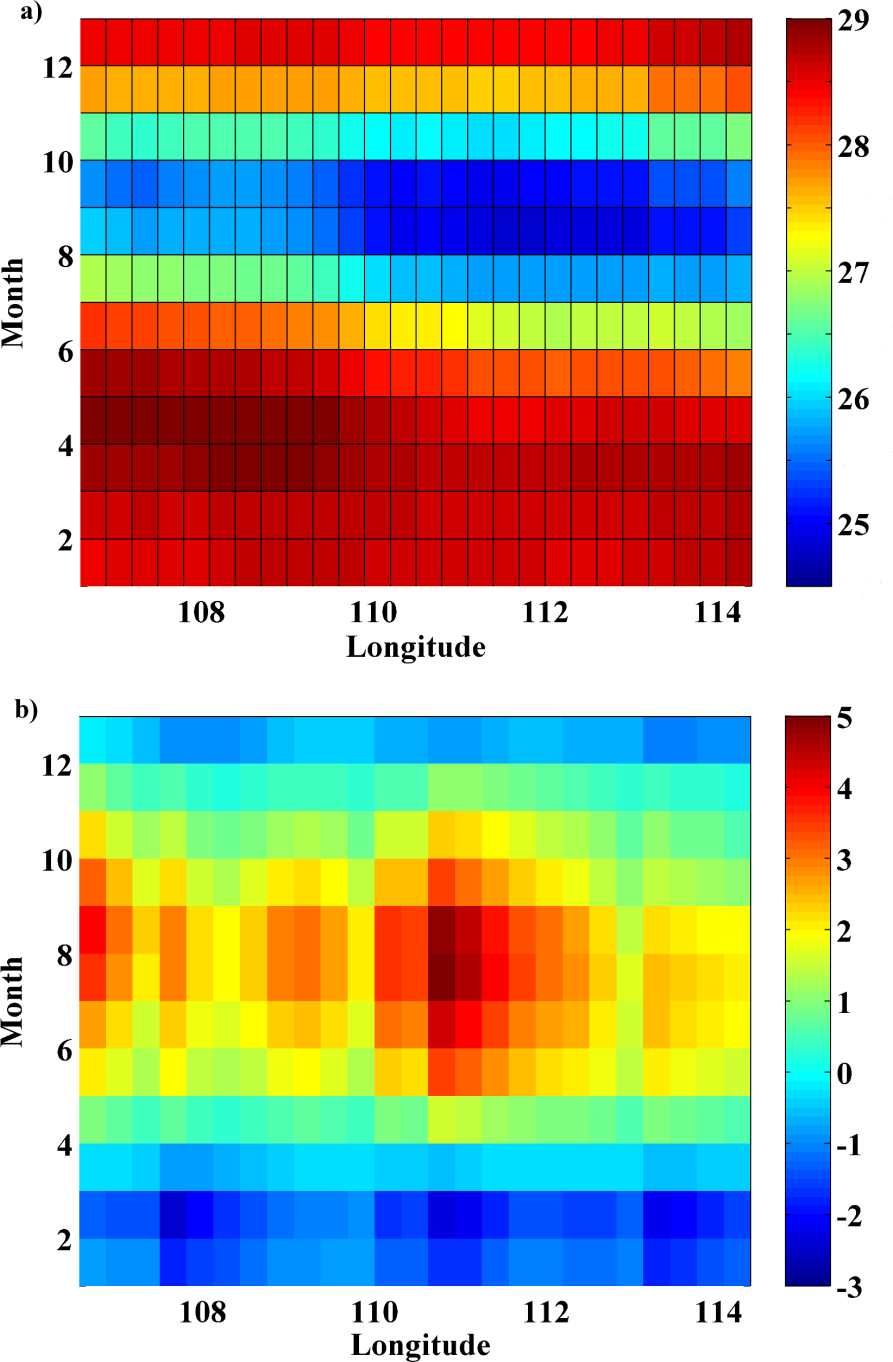
**(a) Annual cycle of SST (**°**C)** along the coast of Java (Fig 1 circles) calculated for the period 1982 to 2015 using OISS ¼ database; **(b) Annual cycle of UI (m**^**2**^**s**^**-1**^**)** along the coast of Java (Fig 1 circles) calculated for the period 1982 to 2015 using CFSR database.

Fig 2 shows the annual cycle of SST (Fig 2A) and UI (Fig 2B) calculated along the coastal points marked in Fig 1 (circles). The lowest SST values (24.5–26.5°C) are detected from July to October (Fig 2A) for almost the entire region. Minimum values are obtained between 110°E and 113°E in accordance with previous works [21]. For the rest of the year SST values are around 27–29°C. The annual cycle of UI (Fig 2B), shows positive values from April to November all along the coast as pointed out in previous studies [16, 31]. The highest values are observed from July to September when southeast monsoon prevails. Considering the UI pattern along the coast, maxima are found from 110°E to 112°E with values up to 4.5 m^2^s^-1^. In contrast, from December to March westerly winds dominate and negative values of UI (around -2.5 m^2^s^-1^) are obtained.

**Fig 3:**
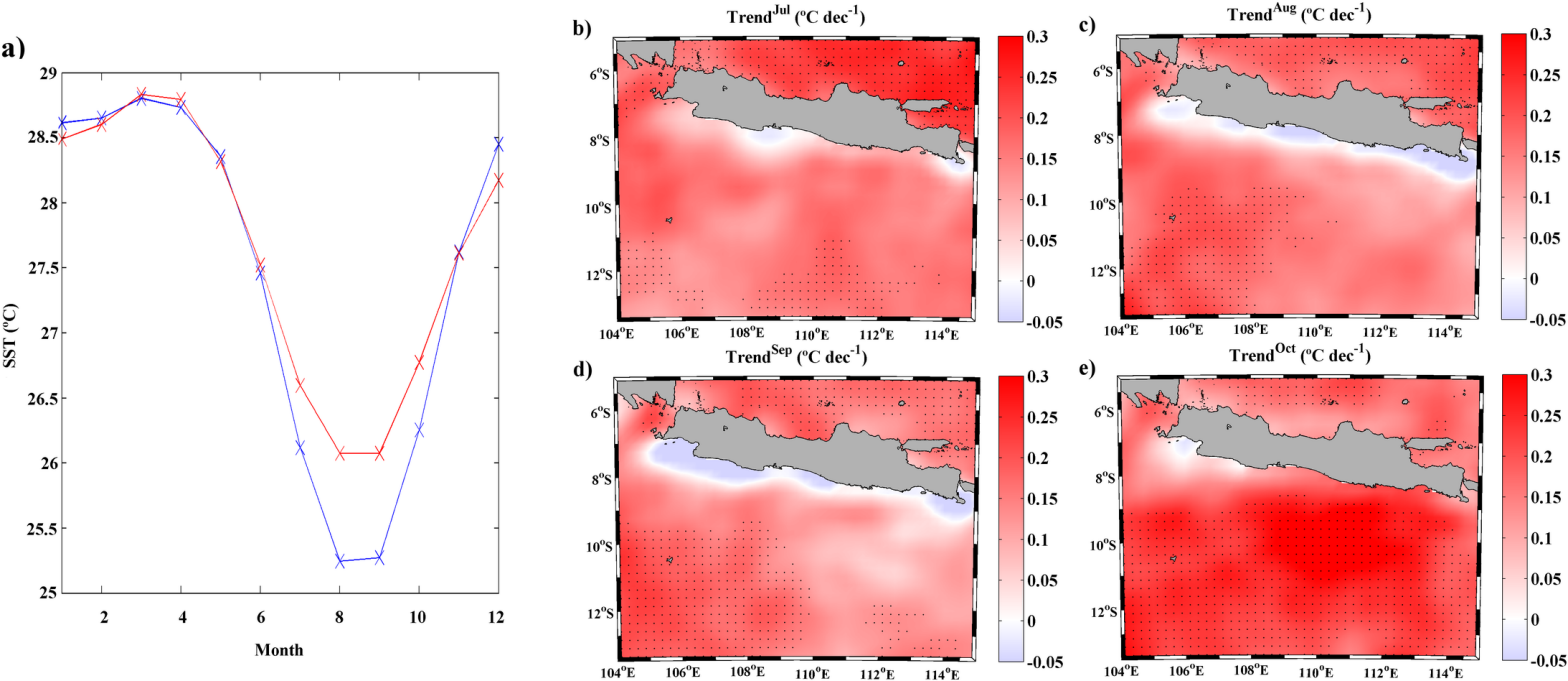
**(a) Annual cycle of SST (**°**C) meridionally averaged** along the coastal (blue line) and ocean locations (red line) for the period 1982 to 2015 using OISST ¼ database. **(b-d) Monthly SST trends (**°**C dec**^**-1**^**)** calculated for the period 1982 to 2015 from July to October using OISST ¼ database.

To analyze the differences in SST at coastal and oceanic locations, the annual cycle of SST meridionally averaged over the points shown in Fig 1, both coastal (circles) and ocean (crosses), was calculated (Fig 3A). From November to June coastal and oceanic SST values remain very similar, while from July to October differences between coastal and oceanic points are clearly detected. The highest differences (~1°C) occur between August and September when UI presents higher values (Fig 2B). This different behavior between coastal and oceanic locations indicates a clear influence of upwelling on SST pattern along the coast. Upwelling forcing on SST is an important oceanographic feature in coastal upwelling regions due to the intense pumping of cooler and deeper water to the surface. The importance of this mechanism as a moderator of SST increase has been analyzed by several researchers along different coastal upwelling systems. Thus, Gomez-Gesteira et al. [32] studied the Canary Upwelling System from 1986 to 2006 finding SST differences between coast and ocean on the order of 3°C from August to October linked with the existence of upwelling favorable conditions. Similar results were obtained by Barton et al. [33] for the period 1981–1991 and by Santos et al. [6] from 1982 to 2010. Along the western Iberian Peninsula, Santos et al. [34] found ~1°C of difference between coastal and oceanic locations from 1900 to 2008. Moreover, Santos et al. [7] observed differences between coast and ocean SST values up to 2°C for the period 1900–2009 along the Benguela upwelling system.

The differences in SST evolution at coastal and oceanic locations along the south coast of Java over the last decades of climate change were also evaluated in terms of warming rates. Considering the seasonal differences shown above, the analysis was carried out from July to October. Fig 3B–3E shows the monthly SST trends for the entire region over the period 1982–2015 during these months. A general warming trend is observed for all months especially in the open ocean. The coastal area presents a different behavior with warming rates lower than the oceanic one. In fact, during July and October no tendency is observed along the coast. Only a spot of small negative trends is detected between 107°E and 109°E in July. Focusing on August and September, when UI presents higher values (Fig 2B), a negative trend can be observed all along the coast with values up to -0.05°C dec^-1^. As previously mentioned, Lima and Wethey [3] analyzed changes in coastal SST along this coast using the same database than the present study during 1982–2010. They also reported small negative trends around June and July. It is necessary to take into account that these authors analyzed trends of SST along the south coast of Java as part of the whole Eastern Indian Ocean. Thus, it is difficult to clearly identify the months and coastal zones where negative trends were detected.

**Fig 4:**
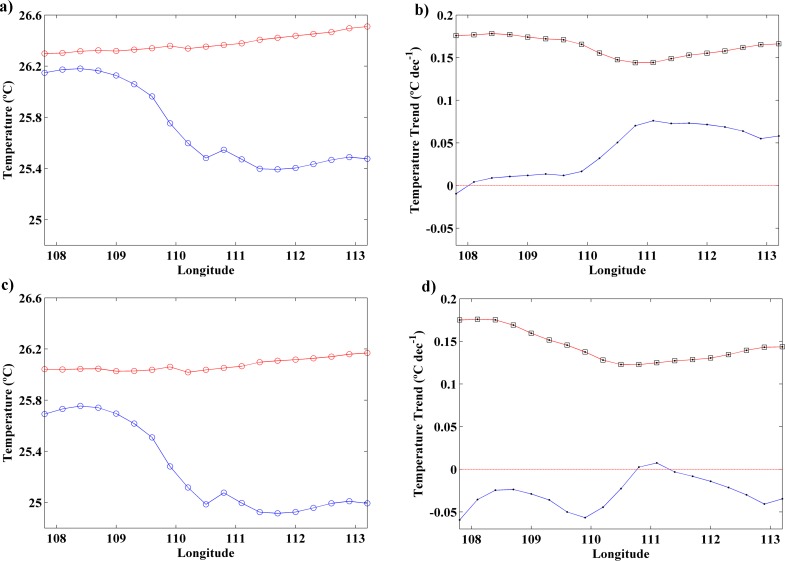
**(a) SST mean (**°**C)** along the coastal (blue line) and ocean locations (red line) from July to October for the period 1982 to 2015 using OISST ¼ database. **(b) SST trends (**°**C dec**^**-1**^**)** along the coastal (blue line) and ocean locations (red line) from July to October for the period 1982 to 2015 using OISST ¼ database. **(c) SST mean (**°**C)** along the coastal (blue line) and ocean locations (red line) from August to September for the period 1982 to 2015 using OISST ¼ database. **(d) SST trends (**°**C dec**^**-1**^**)** along the coastal (blue line) and ocean locations (red line) from August to September for the period 1982 to 2015 using OISST ¼ database. Those points with significance greater than 90% are marked with a circle and those with significance greater than 95% are marked with a square.

A more complete analysis of the differences between the coast and open ocean along the south coast of Java can be observed in Fig 4 in terms of SST means and trends. Fig 4A and 4B (c,d) shows the SST mean and trend from July to October (August-September) for the points located along the coast (blue line) and in the ocean (red line). SST means and trends present higher values at the ocean locations than near the coast for both cases. From July to October (Fig 4A), SST at ocean points presents a similar value all over the region while at coastal locations SST decreases from west to east. The lowest difference (~0.2°C) between coast and ocean locations is observed at the western region. This difference increases eastward reaching a maximum of 1°C around 111°E, which corresponds to the area with stronger UI values (Fig 2B). Analyzing SST trends (Fig 4B), positive values are obtained in both cases (coast and ocean), although the ocean warming rate is higher than the coastal one at all longitudes. The lowest differences between coast and ocean are detected from 111°E to 113°E where ocean and coastal trends are closer. The same analysis was also carried out from August to September (Fig 4C and 4D), the months with the highest difference between coastal and ocean points (Fig 3A). Considering SST means, a pattern similar to the one shown for July-October can be observed with higher values in the ocean locations. Nevertheless, in this case, differences between coast and ocean increase, reaching values around 0.4°C at the western region and 1.2°C at the easternmost longitudes. In terms of SST trends (Fig 4D), the pattern is different from the previous case (Fig 4B). Thus, trend values along the coastal points are mostly negative for the entire region with a difference around 0.25°C dec^-1^ in relation to the ocean.

Previous results have shown that SST trends along the coast can change substantially depending on the different months considered to the analysis. Earlier studies on different upwelling areas showed a high dependence of trends on the length of the time series and even on the season evaluated in the analysis [9, 35–36].

**Fig 5:**
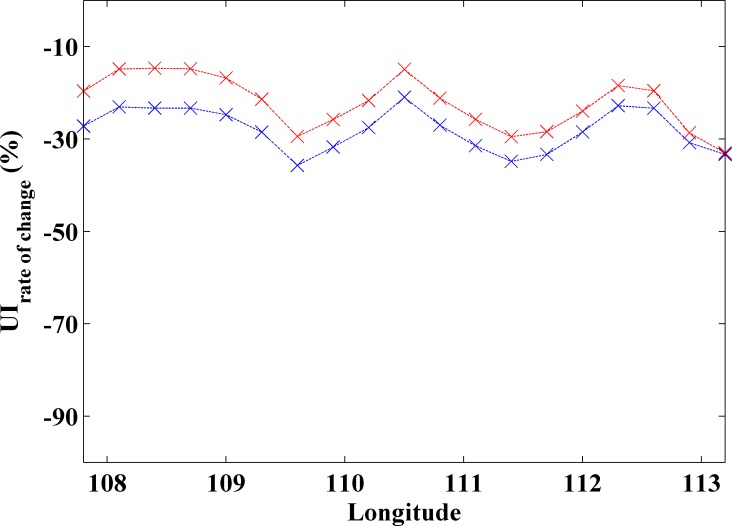
Rate of change of UI (%) along the coastal locations (Fig 1 circles) calculated for the period 1982 to 2015 using CFSR database. Blue line corresponds to July-October and red line to August-September.

As previously mentioned, differences between coastal and ocean warming rates have been previously reported along different upwelling regions as the Western coast of the Iberian Peninsula, the Canary Upwelling system and the Benguela Upwelling System [4–7]. These differences were linked to the strengthening of coastal upwelling acting as a moderator of SST increase. The variation of UI along the last three decades of intense climate change along the south coast of Java is shown in Fig 5. Results are shown from July to October (blue line) and from August to September (red line). The rate of change of UI decreases for both periods, being more negative from July to October with values around 30%. From August to September the rate of change is around 15–20%. These results are in good agreement with upwelling trends recently obtained by Varela et al. [9] in terms of wind stress from 1982 to 2010. They detected a significant decreasing trend for the entire coast considering the upwelling season from May to October.

Results obtained in the present study show that ocean warming is significant and coastal warming negligible during the upwelling season (July to October). In addition, cooling is observed near shore and warming at the ocean during the central months of the upwelling season (August-September). Nevertheless, UI was observed to decrease in both cases. This behavior is different from the one observed at other upwelling regions worldwide [6–9, 35, 37–40], where the different warming rates (sometimes cooling near shore) were due to unchanged or enhanced upwelling scenarios. Upwelling was considered as the main driver in such a way that its mere presence, which pumped cold water up, was enough to hinder surface water warming at the same rate as adjacent ocean water. In those cases where upwelling strengthened, the mechanism became even more efficient and limited warming could turn into cooling as observed in Benguela and La Guajira Upwelling Systems [7, 8].

**Fig 6:**
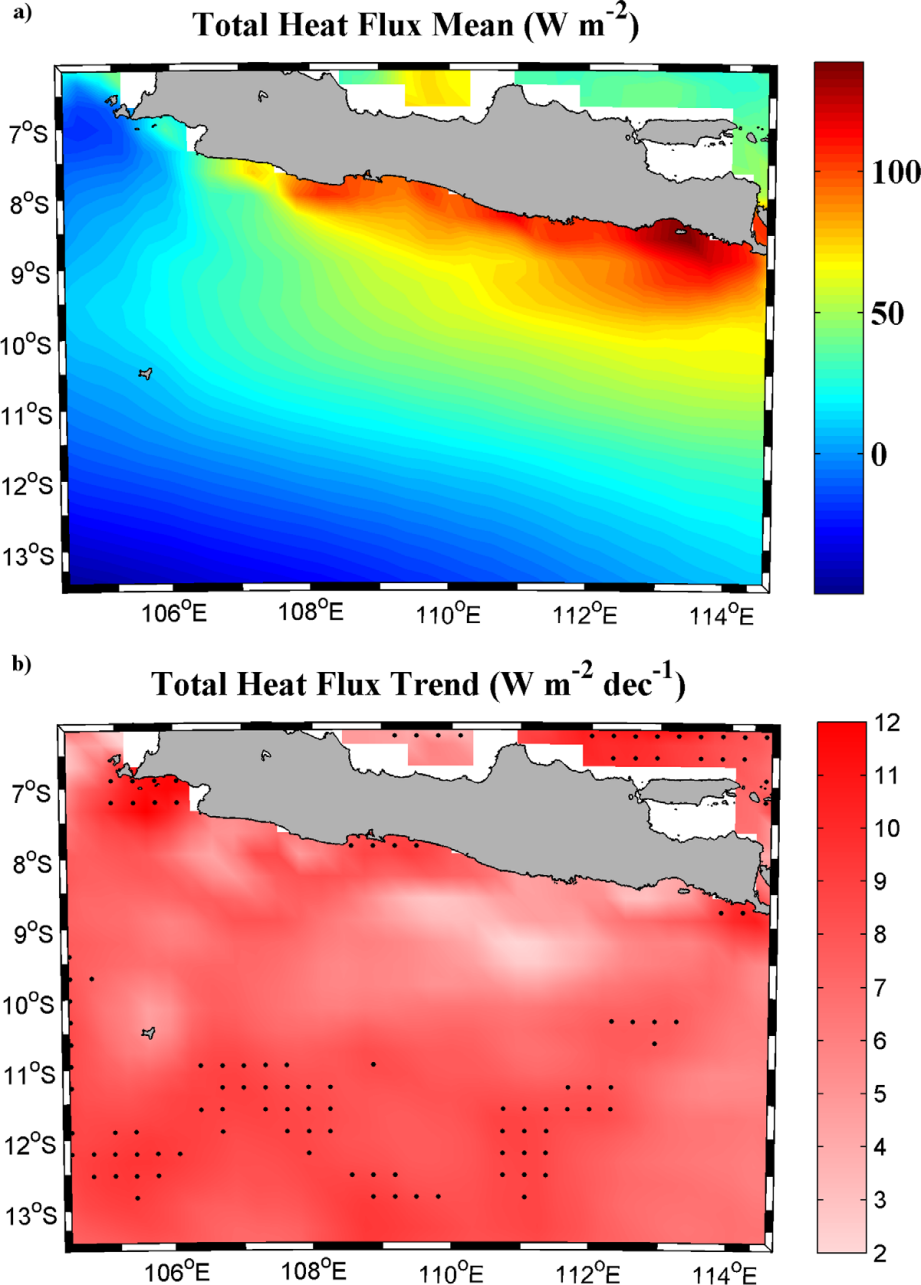
**(a) Heat flux mean (W m**^**-2**^**) and (b) trend (W m**^**-2**^ **dec**^**-1**^**)** calculated over the period 1982–2015 (July-October). Black dots represent grid points with significance higher than 95%. A negative (positive) value implies that ocean is losing (gaining) heat.

**Fig 7:**
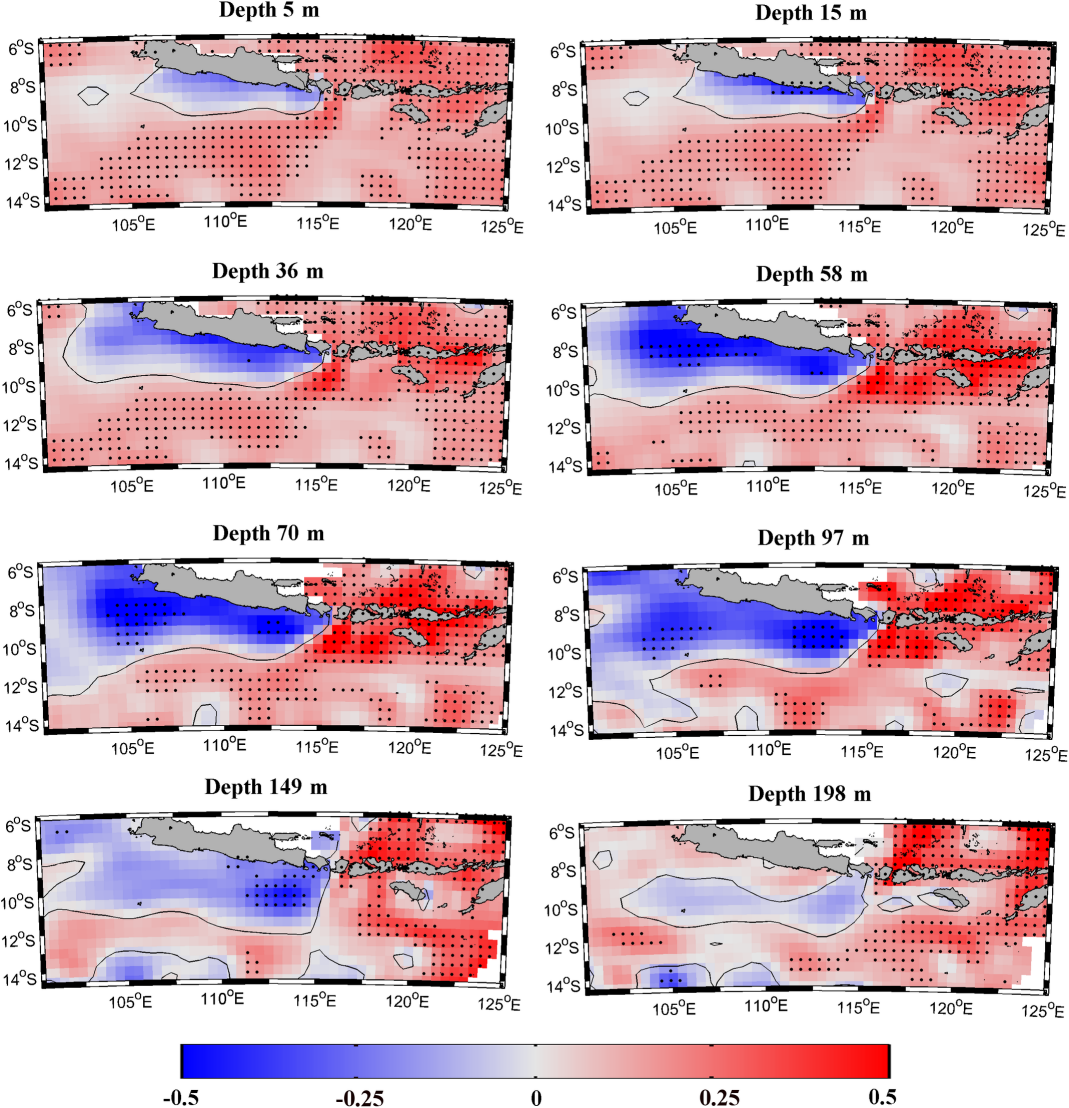
Temperature trend (°C dec^-1^) calculated for the period 1982 to 2010 from July to October at different layers around the south coast of Java using SODA database.

To better understand the apparent contradiction along the south coast of Java, the role of heat exchange between ocean and atmosphere (Fig 6) and the influence of advection processes (Fig 7) were analyzed. Fig 6 shows the mean heat flux and its trend calculated over the upwelling season (July-October). Ocean gains heat (positive values) over a large part of the area under scope (Fig 6A). The highest values are observed nearshore, especially in the central and eastern parts, which coincide with the area where the lowest mean SST values are detected (Fig 1). Total heat flux trends (Fig 6B) show an increase for the whole area with values between 3–12 W m^-2^ dec^-1^. These results suggest that heat exchange between ocean and atmosphere is not the forcing that drives the cooling pattern found in the upwelling area since no differences in heat exchange were detected between coastal and ocean locations.

**Fig 8:**
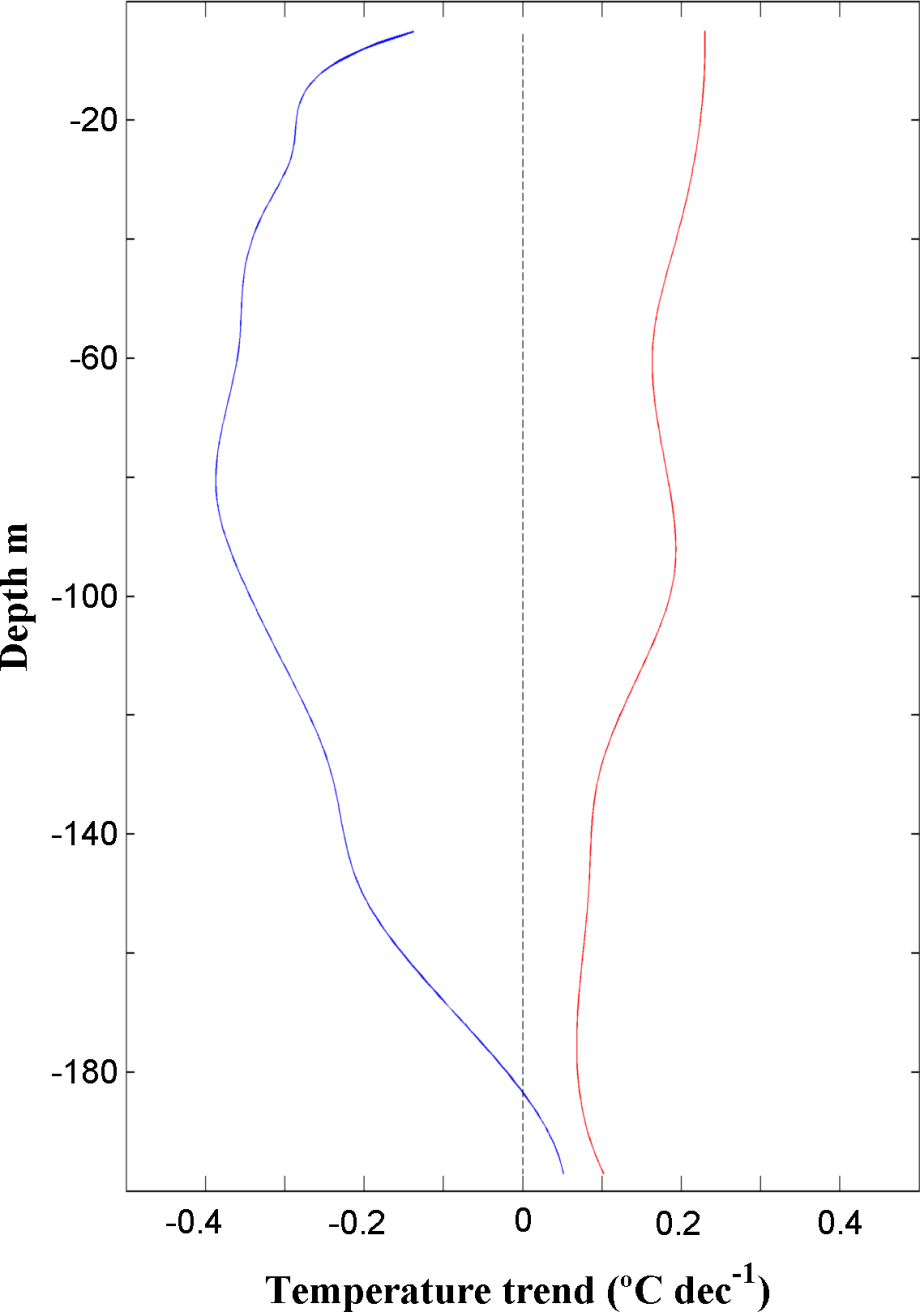
**Temperature trend (°C dec^-1^)** calculated for the period 1982 to 2010 from July to October near coast (blue line) and at the ocean (red line) along the longitude 110°E using SODA database.

Changes in the temperature of the water column (obtained from SODA) were also analyzed as a possible cooling mechanism along the south coast of Java. Fig 7 shows the temperature trends calculated in a wide region around the south coast of Java for eight different layers (from 5 to 200 m) over the upwelling season (July-October). The upper layer (5 m) shows a general warming trend all over the region except along the south coast of Java where a negative temperature trend around -0.1°C dec^-1^ and -0.2C dec^-1^ is observed. This cooling trend spreads to the south and west of the region reaching its maximum extension around 100 m with minimum values around -0.5°C dec^-1^. From then on, the area where the temperature trend is negative becomes smaller with depth and beneath 200 m the negative trend disappears. Note that white regions along the northern coast of Java and at the southeast corner of the map represent no data due to the shallowness. This figure clearly indicates that the water from subsurface layers along the south coast of Java shows a cooling trend over the last three decades. Two vertical sections along the longitude 110°E (Fig 8) were also analyzed using SODA to better analyze the vertical structure of temperature trend from July to October near coast and at ocean locations. Blue (red) line shows the vertical profile calculated near the coast averaging data from 8° to 9.5°S (11.5° to 13°S). Trends are positive at the ocean (red line) decreasing from the upper to deeper layers. As shown in the previous figure, trends are negative near shore (blue line), being the highest value (around -0.4°C dec^-1^) observed between 40–100 m.

In summary, previous results indicate that the role of heat exchange between ocean and atmosphere is negligible to explain the different warming patterns observed at coastal and oceanic areas when compared with ocean processes. Thus, horizontal advection (cool water advected by the South Java Current [41, 42]) and vertical entrainment due to upwelling are the main forcings that drive cooling off Java. In spite of the moderate decrease in UI, the combination of both processes is still efficient to bring cooled water to the surface.

## Conclusions

Within a context of climate change with a general upward trend in terms of SST, the south coast of Java has shown a different behavior over the last three decades (1982–2015). A small coastal warming was detected over the upwelling season (July-October) along with a moderate decrease in UI. This behavior contrasts with that observed in other upwelling regions as Benguela, Canary or La Guajira upwelling Systems where a moderate coastal warming, or even cooling, was linked to the strengthening in coastal upwelling.

The analysis of the heat exchange between ocean and atmosphere showed that this forcing was not the responsible of the cooling trend found in the upwelling area. In fact, the vertical structure of water temperature along the south coast of Java showed that subsurface layers have experienced a cooling trend over the last three decades. Thus, although UI presented a negative trend, it can still pump cooled water to the surface.

Trends shown in this work confirm the interest of studying local areas where upwelling is an important forcing, taking advantage of the high spatial resolution of databases to resolve conditions at the scale of coastal upwelling. The obtained results also showed the interest of analyzing the properties of subsurface water masses, especially in areas where they can be brought to surface by upwelling.
